# Molden 2.0: quantum chemistry meets proteins

**DOI:** 10.1007/s10822-017-0042-5

**Published:** 2017-07-27

**Authors:** Gijs Schaftenaar, Elias Vlieg, Gert Vriend

**Affiliations:** 1 CMBI, Radboudumc, Nijmegen, The Netherlands; 2 Institute for Molecules and Materials, Radboud University Nijmegen, Heyendaalseweg 135, 6525 AJ Nijmegen, The Netherlands

**Keywords:** Molecular visualisation, Molecular modelling, Quantum mechanics, Electrostatic potential, Electron density, Protein manipulation

## Abstract

Since the first distribution of Molden in 1995 and the publication of the first article about this software in 2000 work on Molden has continued relentlessly. A few of the many improved or fully novel features such as improved and broadened support for quantum chemistry calculations, preparation of ligands for use in drug design related softwares, and working with proteins for the purpose of ligand docking.

## Introduction

The Molden software was conceived in the early 1990s and first published in 2000 [1]. Molden 1.0 was designed as a tool to support quantum chemistry calculations by pre-processing input data and by visualizing the computational results. Manual inspection of a few hundred of the articles that cite Molden 1.0 revealed that it is used most often for visualisation of wave-functions, for construction and editing of molecules via its Z-matrix functionality, and for validating stationary point(s) at potential energy surfaces. Most citations came from labs working in the drug design field. Surprisingly, we also found a large number of examples of mutations of amino acids (e.g. [2–4]). Molden has been cited more than 2000 times according to the Web of Science [5], but very many more times according to Google Scholar. Registered Molden users now exceed 15,000; including most large pharmaceutical industries.

Molden was initially designed to augment computational work in quantum chemistry by providing visualisation facilities, and by dealing with data formats and other administrative tasks. Indeed, it became so useful in the quantum chemistry field that its wave-function format that was designed to interface with quantum mechanics packages is now a de facto standard [6, 7]. Table 1 summarizes some popular facilities (as judged by the number of citations) that were available already in Molden 1.0, and that have been further developed over the years without modifying their goals or fundamental concepts.

**Table 1:** Selection of popular Molden 1.0 facilities that were recently improved

Display molecular density from the ab initio packages Gamess-US, Gamess-UK, Gaussian, and the semi-empirical packages Mopac and Ampac	Rudimentary support for protein visualisation
Display molecular orbitals, electron density, and difference density	Fitting atomic charges to the electrostatic potential calculated on a Connolly surface
Calculation and display of the true and multipole derived electrostatic potential	Calculation and display of the Laplacian of the electron density
Animation of reaction paths and molecular vibrations	Additional support for a number of other QM packages via the Molden format
Versatile Z-matrix editor	Support for crystal visualisation

Computers are today so fast that quantum chemical calculations are becoming accessible to everybody, and our inspection of articles that cited Molden revealed that quantum chemistry is becoming a frequently used tool in fields like drug design [e.g. 8–11], nanoscience [e.g. 12, 13], and organometallics [e.g. 14]. The scope of Molden was therefore broadened to also support a whole series of activities commonly employed by computational chemists working in the pharmaceutical industry. Table 2 lists a few of these new facilities, some of which will be discussed more extensively in the "Results".

**Table 2:** Selection of novel facilities in Molden 2.0

Display molecular density from the ab initio packages ORCA, Nwchem, and QChem.	Calculation and display of localized molecular orbitals and the electron localisation function.
Display solvent-accessible surfaces optionally with electrostatic potentials mapped onto it	An amino acid sequence editor to create three dimensional peptides
Visualisation of NMR and UV spectra	Interactive docking with PMF scoring
Energy minimisation program Ambfor for geometry optimisation with the combined Amber (protein) and GAFF (small molecules) force fields	Stand-alone molecular dynamics program Ambmd using the Amber (protein) and GAFF (small molecules) force fields
Rotamer space scanning to probe active site flexibility	Extension of the Z-matrix editor to include protein editing
Adding hydrogens to ligands and proteins in a PDB file	A small molecule crystal optimiser
A residue command window to turn the visibility of residues/ligands off/on	Handling multiple protein structures and allow users to align or superpose them
A native Windows version is available that does not require the Xwindows emulator, but makes use of the OpenGL graphics library [16]	Support for QM calculations making use of pseudo potentials

Molden has been distributed more than 15,000 times to registered users, but the number of downloads is a multitude of this number. We answer on average 3.2 user questions each day. We intend to support Molden for at least ten more years. It is available for the operating systems Linux, Windows, and OS X. It can be downloaded from ftp://ftp.cmbi.ru.nl/pub/molgraph/molden/. Both tarred and gnu-zipped source code are available as well as binaries for these three operating systems. The website (http://www.cmbi.ru.nl/molden/) lists all the latest software developments and provides a series of help facilities. This includes a manual with a description of the keywords to control Molden through a keyword file.

## Methods

The Molden code consists of 100,000 lines Fortran and 60,000 lines C. The C code allows for the efficient communication with graphics application programming layers (API’s). Molden makes use of two such API’s: The X Window System protocol client library [15] and the Open Graphics Library (OpenGL) [16] that is a multi-platform API for rendering 2D and 3D vector graphics. In addition, Molden uses the OpenGL Shading Language (GLSL) [17] to program shaders, small programs that run on the graphics hardware to produce special effects such as per-pixel lighting, blurring, shadows, and ambient occlusion. The native Microsoft Windows version of Molden uses the Simple DirectMedia Layer (SDL2.0) [18] to replace the X window library.

Molden’s ‘read file’ window, allows for the direct download (using the external network download program ‘wget’) of files from the PDB (http://www.rcsb.org/) by specifying the unique four-letter PDB-identifier. The same ‘wget’ can also be used to retrieve information about missing hydrogens from the EBI’s PDBECHEM database [19].

## Results

Most new facilities fall in one of three categories: support for quantum chemistry, support for work with ligands, and support for working with proteins (see Table 3).

**Table 3:** Novel Molden facilities mainly fall in one of three groups

Quantum chemistry^a^
QM package support
Localised orbitals
Electron localisation function (ELF)
Visualisation of spectra
Working with ligands^b^
Polar surface area (PSA)
Alignment of molecules
Crystal optimiser based on the gaff force field
Interactive docking with potential of mean force (PMF) scoring
Partial optimisation of a protein–ligand complex
Working with proteins^c^
Protein editing via the z-matrix
Rotamers: editing/search rotamer space
Optimisation of hydrogen positions
Display of protein electron density maps
Ambfor and ambmd: protein geometry optimisation and protein dynamics
Addition of hydrogens to ligands
Fixing incomplete residues: missing side-chain atoms of amino acids can be added
Protein-specific visualisation facilities

This table is not exhaustive. More information can be found in the documentation at the Molden home page: http://www.cmbi.ru.nl/molden/. All facilities mentioned in this table are explained in the remainder of this article

^a^Support for quantum chemistry calculations

^b^Support for ligand preparation

^c^Support for working with proteins for the purpose of ligand docking

### Quantum chemistry

#### QM package support

Molden could already parse output of the quantum chemistry (QC) packages: Gaussian [20], Gamess-US [21], Gamess-UK [22] and Mopac [23], and this list was recently extended with NWchem [24], Orca [25] and Qchem [26]. The Molden format was developed to interface to QC programs that produce output that cannot be parsed natively. This format includes all information required for orbital/density and molecular vibration visualisation (e.g. Cartesian coordinates, the basis-set, the molecular orbital coefficients and occupancy numbers). The Molden format (see http://www.cmbi.ru.nl/molden/molden_format.html for a description) is currently used by a series of prominent QC programs (see Table 4).

**Table 4:** Packages that produce Molden format files

Package	URL
ACES II	http://www.qtp.ufl.edu/
MOLCAS	http://molcas.org/
MOLPRO	http://www.molpro.net/
DALTON	http://daltonprogram.org/
JAGUAR	https://www.schrödinger.com/

Molcas [27] is a popular ab initio computational chemistry program that focusses on the calculation of electronic structures in ground and excited states. The Molcas authors recommend that their users apply Molden for visualization. The widely used and often cited Gabedit software [28] (freeware) is a graphical user interface, offering pre-processing and post-processing options for nine computational chemistry software packages. The Molden format has a prominent position in their list of supported formats. So they use Molden to widen their application base.

#### Localised orbitals

**Fig. 1:**
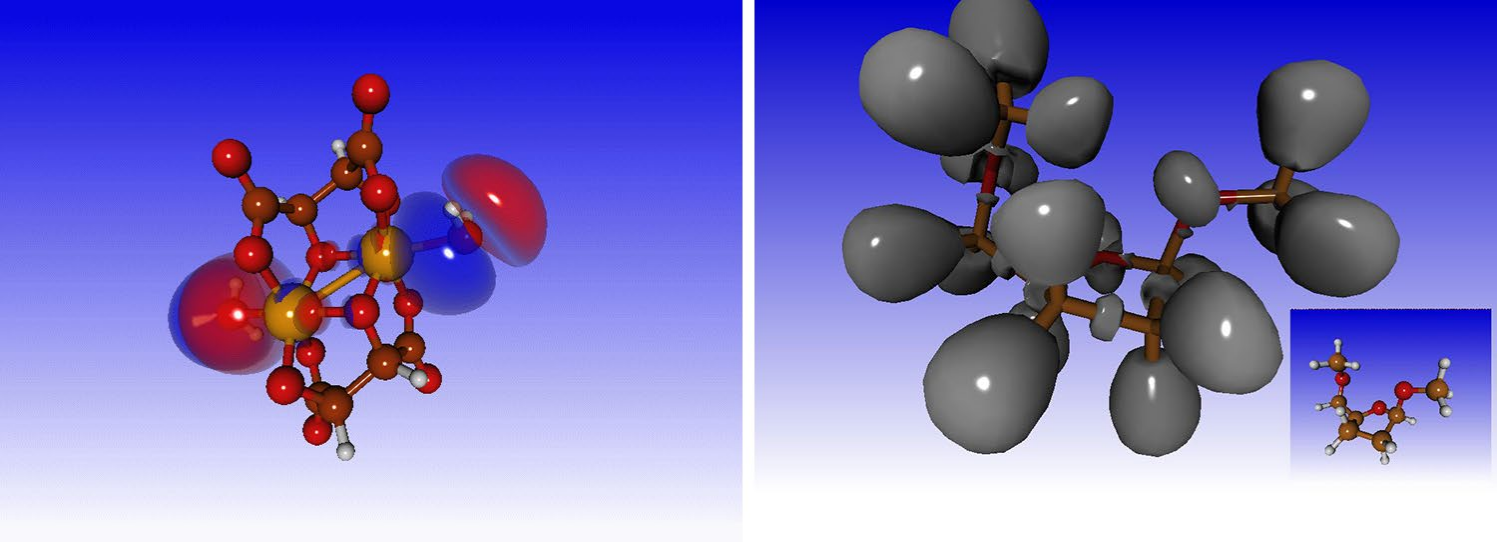
Orbital visualisation. Standard ab initio quantum chemistry methods yield delocalised orbitals that extend over the entire molecule. Localised orbitals can be found as linear combinations of the occupied delocalised orbitals by a unitary transformation. In Molden the Foster-Boys [29] scheme is employed to localize molecular orbitals. The *left-hand panel* shows the localised orbital of Iron(III) meso tartaric acid. This calculation proves that there is a bond between the irons and the coordinated water molecules (ultimate *left* and *right* in the figure). The absence of nodal planes between iron (*yellow*) and water(s) is tantamount to the presence of electron density (a bond) between irons and water. The right-hand panel shows an example of the ELF on 2,5 Dimethoxyfuran with evidence of lone pairs and covalent bonds. *Inset* a ball-and-stick representation of the molecule (carbon in *brown*, oxygen in *red*, hydrogen in *white*)

Visualisation of orbitals is an important aspect of many types of research. A series of orbital visualisation options, such as localised orbitals and electron localisation functions are provided (Fig. 1 illustrates a few of the orbital visualisation options).

Masunov et al. [29], for example, introduced a new method to eliminate the spin-contamination in broken symmetry density functional theory calculations. They investigated two complexes of which one had strongly localized magnetic orbitals on which previous spin-contamination eliminate schemes worked well. The other complex had strongly delocalised magnetic orbitals on which previous schemes failed while their new scheme works well. Both restricted and unrestricted natural orbitals were visualized with the help of Molden.

In his landmark article on the free radical catalysis by galactose oxidase, Whittaker [30] used Molden to calculate visualise the crucial SOMO (Singly occupied molecular orbital) to determine at which atom the radical electron is located.

For a newly designed anilate-based material with luminescence properties, for example, the electrostatic potential calculated by Molden was used to strengthen the conclusions from the analyses of the atomic charges [31]. Atzori et al. wrote: “The isodensity surface mapped with the electrostatic potential shows for all systems that the oxygen atoms are the source of greater negative charge accumulation followed by the nitrogen atom of the CN moiety. Moreover, there is a moderate negative charge accumulation on the carbon atoms linked to the Cl and CN groups, whereas the remaining four carbon atoms, which are linked to the oxygen atoms, exhibit a positive charge. The chlorine atoms present a typical positive charge on the opposite side of the C–Cl vector and a ring of negative charge perpendicular to the same vector” [32].

Hunt et al. [33] used Molden’s electron density map and Laplacian contour facility to examine charge densities, natural bond orbitals, and delocalised molecular orbitals in ionic liquids to explain the relative acidity of different sites on the imidazolium ring and variation in hydrogen-bond donor and acceptor propensities.

#### Electron localisation function

The Electron Localisation Function (ELF) [34] is a measure for the probability of finding an electron with the same spin in the neighbourhood of a reference electron at a given location (see Fig. 1). The ELF shows clear separation between core and valence electrons, and also shows covalent bonds and lone pairs. Whereas the electron density decreases monotonically with the distance from the nucleus, the ELF illustrates the shell electronic structure (S, P, and D shells) of the heavy atoms as clear maxima and minima.

#### Visualisation of spectra

**Fig. 2:**
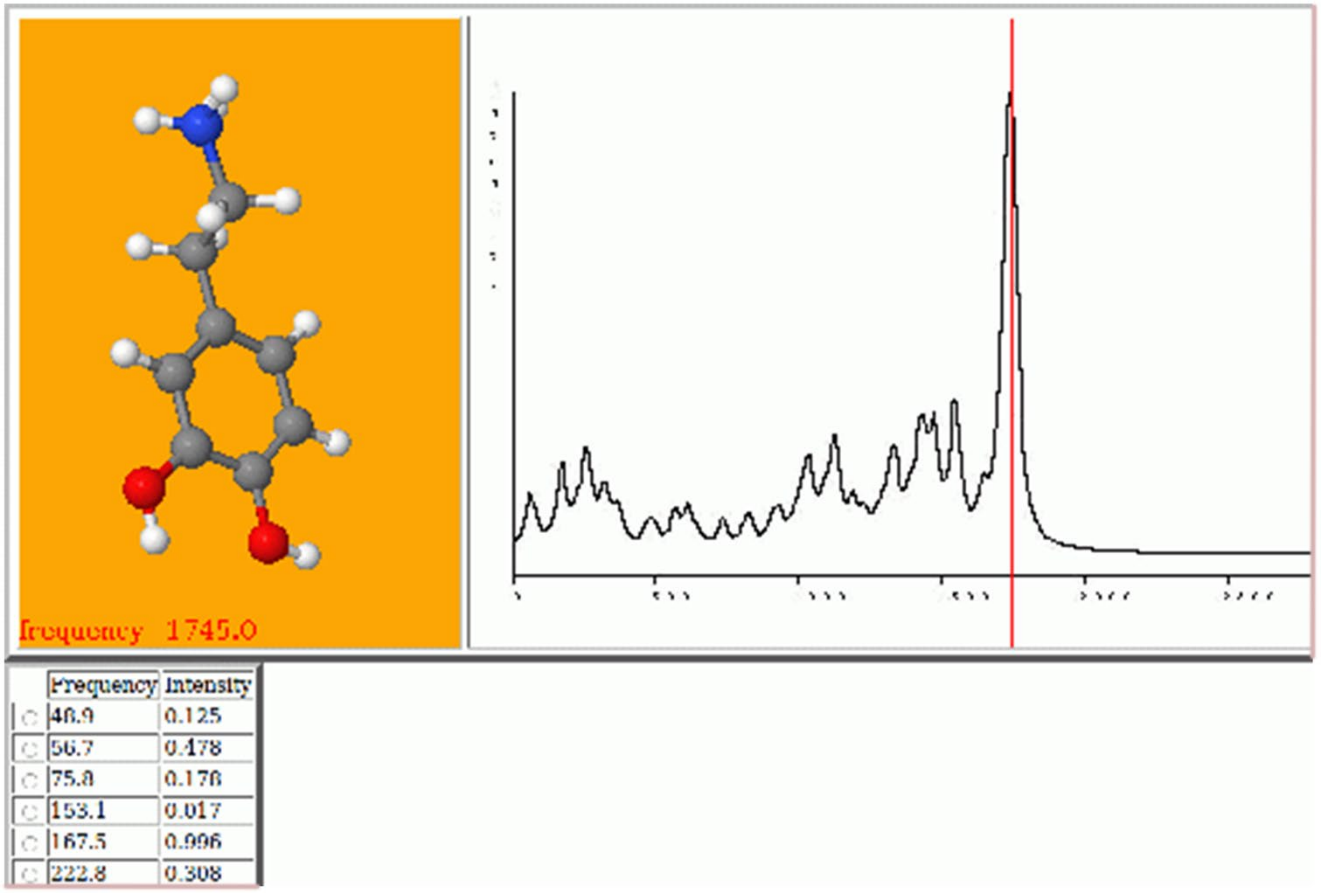
Interactive spectrum as .html document. Clicking on a peak in the spectrum or in the table underneath the spectrum results in the animation of the associated molecular vibration in the molecular display to the *left*

Visualisation of infrared and Raman spectra was already in place at the time of the first Molden paper. This functionality has been expanded with the option to create a html page and auxiliary files, containing an interactive spectrum in combination with an animation of the selected vibration with the jmol viewer [35] An example is shown in Fig. 2; see also http://wetche.cmbi.ru.nl/ calspec/database/0000004/. A.jdx file of the spectrum is written for use with the jspecview program [36]. UV-spectra are constructed and visualized from TD-DFT calculations with Gaussian. ^1^H and ^13^C NMR spectra are constructed and visualized when magnetic shielding and J-coupling information is available from the Gaussian output. With a click on the ‘J’ button, the J-coupling between two selected atoms is displayed. The magnetic shielding and J-coupling corresponding with rotationally equivalent hydrogens can be averaged interactively.

Vidal-Iglesias [37], for example, made assignments of the calculated frequencies of monolayers of 4-aminobenzenethiol (4-ABT) on copper, based on the visualisation of the vibrational normal modes and the Surface-Enhanced Raman spectrum (SERS) using Molden. They write “Surface-enhanced raman scattering (SERS) spectra of self-assembled monolayers of 4 aminobenzenethiol (4-ABT) on copper (Cu) and silver (Ag) surfaces decorated with Cu and Ag nanostructures, respectively, have been obtained with lasers at 532, 632.8, 785, and 1064 nm. Density functional theory (DFT) has been used to obtain calculated vibrational frequencies of the 4-ABT and 4,4′-dimercaptoazobenzene (4,4′-DMAB) molecules adsorbed on model Cu surfaces.”

### Working with ligands

#### Polar surface area

**Fig. 3:**
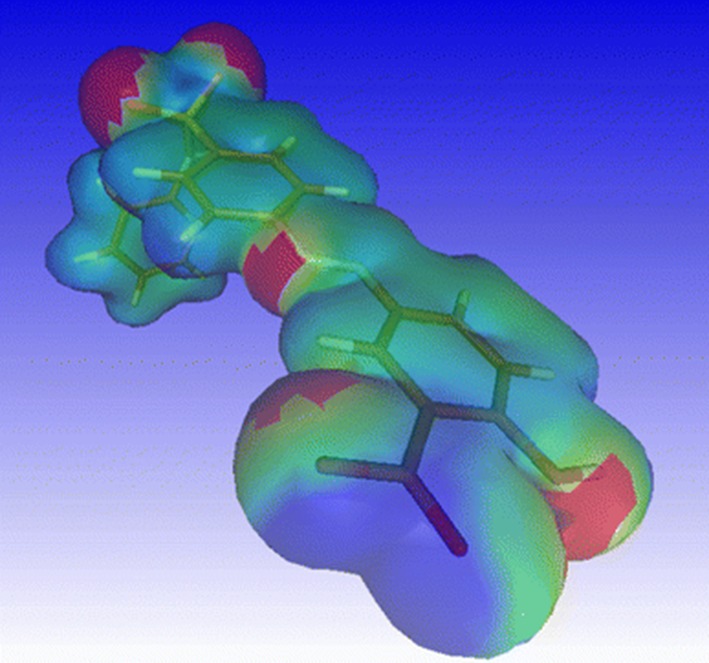
QMPSA. Quantum mechanical polar surface area for the drug molecule sulfasalazine. *Red* and *blue*: polar; *green*: apolar surface area

The polar surface area (PSA) is defined as the combined surface area belonging to oxygen and nitrogen atoms and their hydrogen atoms. Palm et al. [38] were the first to use a calculated PSA to predict the absorption of drugs. A new method was designed to derive the PSA by quantum chemical means QMPSA [39]. This is illustrated in Fig. 3. The original method by Palm et al., and our QMPSA have both been implemented in Molden.

Ren et al. [40], for example, analysed anticancer fungal polysaccharides based on physiochemical properties and identified a unique region in chemical space using a series of molecular descriptors including Molden’s QMPSA.

#### Alignment of molecules

**Fig. 4:**
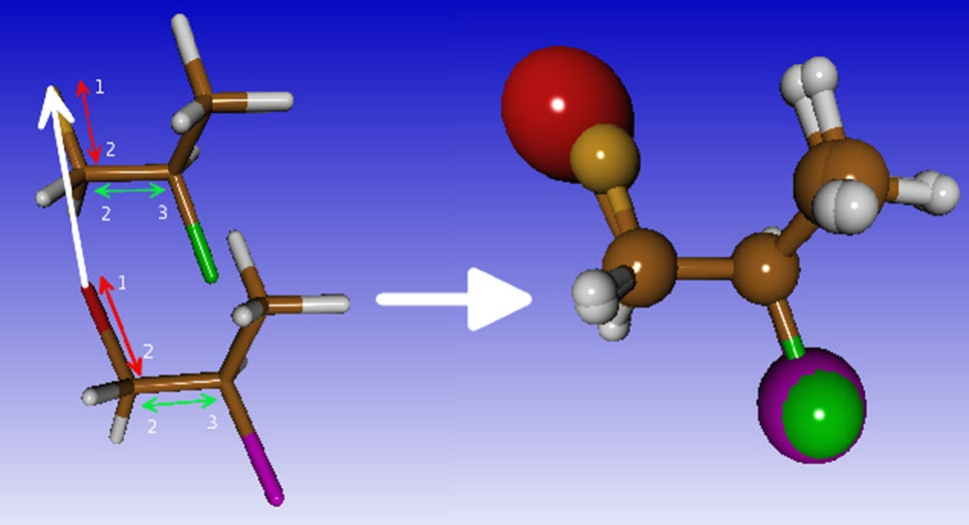
Alignment of small molecules. Three equivalent atoms are selected for each molecule. The atoms labelled 1 are used to translate the first molecule on top of the second. The vectors from atom 1 to atom 2 and atom 2 to atom 3, respectively, are used for two consecutive rotations. The user can select any three atoms

Alignment of molecules -also known as structure superposition- has been implemented following two separate strategies, one for small molecule alignment and one for the alignment of proteins. The alignment of small molecules is illustrated in Fig. 4.

#### Crystal optimizer based on the small molecule gaff force field

Molden 1.0 was already able to read a number of file formats containing crystal information (such as the FDAT and chemx formats) and it was able to display the crystal as a number of unit cells along one or more of the cell axes. The possibility to edit unit cell constants a, b and c, and angles α, β and χ and space group was added later, as well as the possibility to rotate the atoms in the unit cell. In Molden 2.0 the capability to optimise the crystal geometry was added.

**Fig. 5:**
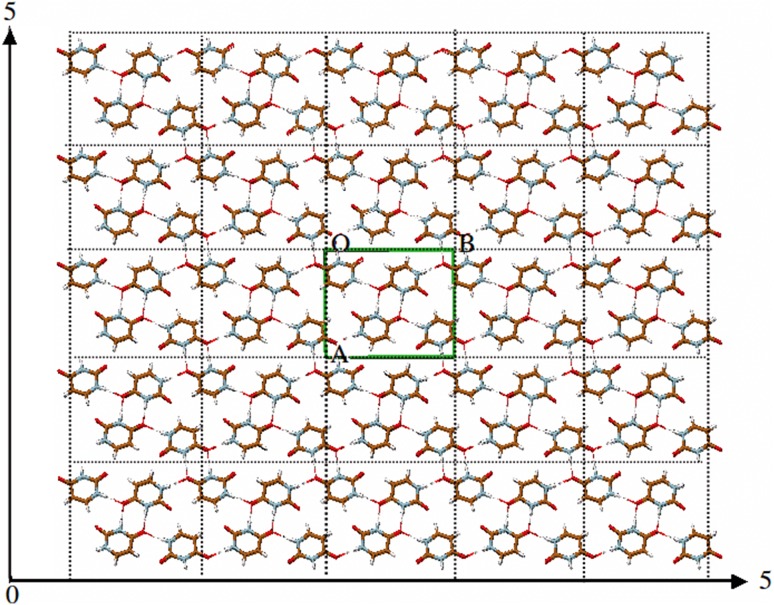
Crystal optimisation. Approximation of lattice sums by calculating all pairwise interactions in a 5 × 5 × 5 super cell expansion of the unit cell (*green*).For clarity only two dimensions are shown

The crystal is computationally approximated by a 5 × 5 × 5 grid of copies of the unit cell (in green) placed at the centre of the grid (see Fig. 5). Neutral charge groups are employed by summing the long-range electrostatic interactions between the molecule(s) in the unit cell and its copies on the 5 × 5 × 5 grid. The geometry of the molecule(s) in the unit cell and the lattice parameters can be optimized using the small molecule force field GAFF [41] and a Powell-Beale conjugate gradient scheme [42]. The GAFF force field requires that partial charges are assigned using a restrained electrostatic potential fit (RESP) model [43]. Other, simpler charge models are available in Molden too. These may be used when very accurate energy calculations are not required. A parallel implementation of the crystal optimizer is available.

#### Ligand docking with PMF scoring

Molden facilitates both interactive docking and fully automatic optimisation of ligand protein complexes. For interactive docking a Potential of Mean Force (PMF) scoring function by Muegge and Martin [44] is being used, while the automatic optimisation of ligand protein complexes uses the AMBER force field [45]. The PMF is derived from the radial distribution of distances between atoms of two distinct types from the PDB database (the available atom types are listed in the Molden documentation). Muegge and Rarey have reviewed the PMF scoring function in comparison to other scoring functions [46] and reported that the PMF score outperformed the energy score and the empirical score of FlexX and is less sensitive to small coordinate changes than the FlexX score. The PMF score was the only scoring function for which a statistically significant correlation could be found between the predicted score and the measured binding affinities of inhibitor-ligand complexes. A comparison for a variety of sets of protein–ligand complexes from the PDB showed the superiority of PMF scoring over SMoG and Böhm’s score.

**Fig. 6:**
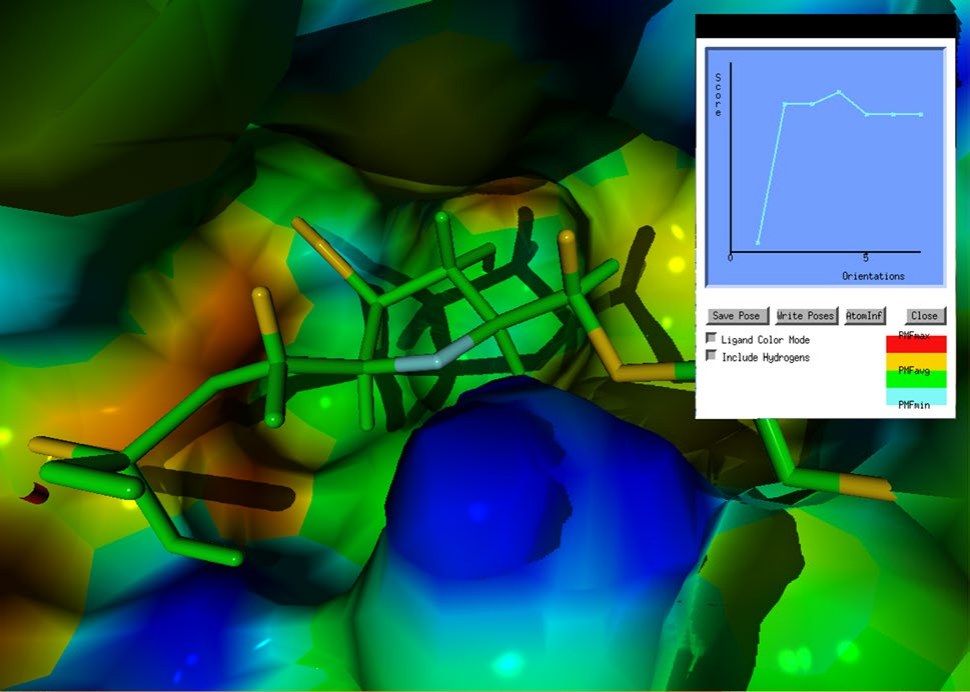
Interactive docking of a ligand. The *inset pop-up window* shows the PMF score

The PMF distributions are converted to the interatomic energy function [47]. The PMF score is used by Molden as a measure of the likelihood of a particular ligand–protein conformation. Conformations can be generated interactively by rotation and translation of the ligand with respect to either the protein, or the world-view. Scores are displayed in a dedicated window that is continuously updated. Individual high/low scoring atom pairs can be highlighted. Figure 6 illustrates Molden’s interactive docking facility.

#### Partial optimisation of a protein–ligand complex

Molden can also perform an AMBER based optimisation of either the whole protein–ligand complex, or of any user-selected part of it. In the latter case, the input of the user is limited to selection of the residues near the ligand as flexible or rigid, using a pop-up window dedicated to this task.

### Protein support

Molden 2.0 has a series of facilities built-in to support working with proteins when docking ligands. These options are directed towards visualisation of proteins and protein–ligand complexes, determining alternate pocket conformations, the optimisation of protein structures or protein–ligand complexes, and towards the actual ligand docking process itself.

#### Protein editing via the z-matrix

**Fig. 7:**
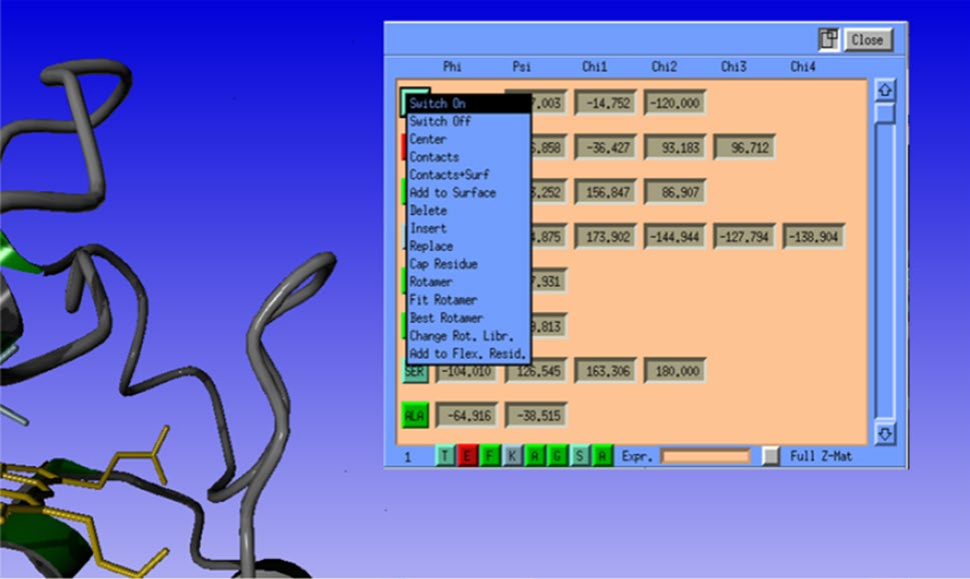
The Z-matrix editor pop-up window. Each residue is represented by a column with a button labelled with its three letter amino acid code and entry fields for the φ, ψ, ω angles. Clicking the button brings up a pop up menu containing residue manipulating options

The Z-matrix provides an alternative to specifying a geometry by Cartesian coordinates (see Fig. 7). In the Z-matrix approach, atom positions are defined with respect to previously defined atoms by means of internal coordinates such as bond distances, bond angles and dihedral angles. For small molecules a Z-matrix can often be constructed ‘by hand’, but for larger molecules this quickly becomes tedious and complex. The impractically large number of variables in a Z-matrix of a protein necessitates a dedicated Z-matrix view of only the most important internal variables, such as φ, ψ, ω, χ1. After interactive selection of an amino acid, the amino acid manipulation menu pops up. From this menu several manipulations can be performed, such as mutation to another amino acid, deletion or insertion of an amino acid, or changing the amino acid’s rotamer. These options are realised by Z-matrix manipulation.

#### Rotamers: editing/search rotamer space

**Fig. 8:**
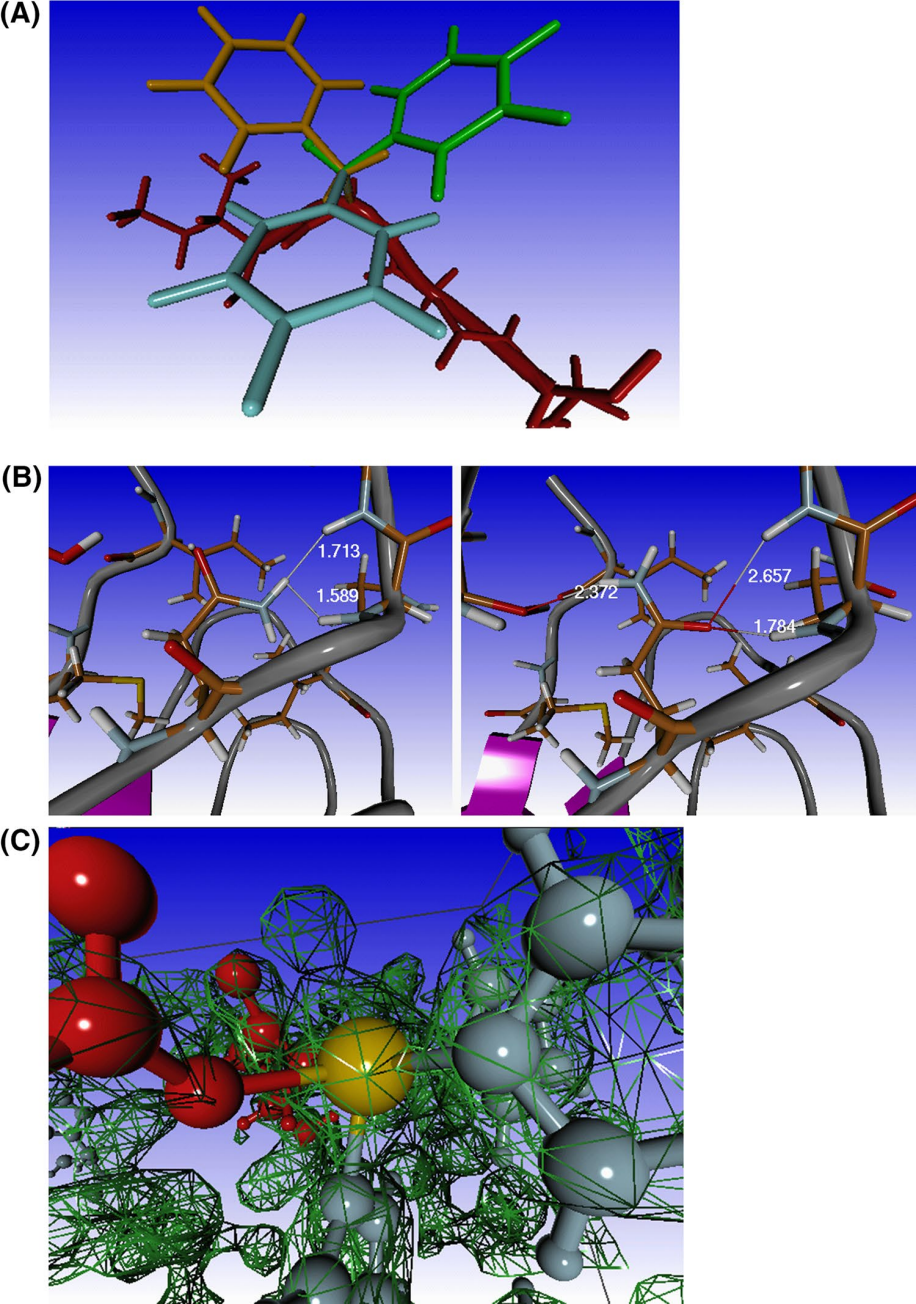
Protein visualisation options. **a** Three of the six rotamers of the residue phenylalanine, indicated by colours *blue, orange* and *green*. **b** PDB entry 1REI, (*left*) residue Gln90 un-flipped and (*right*) flipped. The un-flipped situation shows an energetically unfavourable close contact (in *white* numbers) between two hydrogens. **c** Display of electron density for PDB file 2ETE at contour level 2.0

Amino acid side chains often have several possible conformations commonly known as rotamers. The local structure and a series of external factors (solvent related like pH or salt concentration, the presence of bound ligands or bound ions, the multimeric state of the protein, etc) will influence how often each rotamer is observed. Amino acids tend to prefer rotamers angle is near any of these three values, the rotamer is called gauche-, gauche+, or trans, respectively [48]. Depending on number of rotatable bonds in the amino acid side chain, residues can have from only one rotamer (Gly, Ala) up to 81 rotamers (Lys). In Molden, rotamers are available from either the Richardson [49] or the Dunbrack [50] rotamer library. Molden can scan a part of a protein’s rotamer space (up to a maximum of six residues at a time). This can be instrumental in finding the lowest energy rotamer combination, after a particular residue has been mutated/substituted. The initial scoring is done via the Dfire PMF score. The best ten rotamer combinations will be remembered. The rotamer combinations can be rescored with the AMBER force field. While performing a rotamer scan, Molden will try each conformation available in the rotamer library for the residues considered in the scan, and it will search for the best rotamer by determining DFIRE PMF [47] scores. Substituting a small residue by a bulky one, the surrounding residues are allowed to adopt their rotamer in order to make room for the bulky side chain. An example of the scanning of the rotamer space of a phenylalanine residue is shown in Fig. 8a.

#### Optimisation of hydrogen positions

It is hard to experimentally determine the positions of hydrogens in protein structures. Consequently, hydrogen positions must be determined computationally. Optimisation of hydrogen positions includes optimisation of the hydroxyl orientation of threonine, serine, and tyrosine residues using the AMBER force field for scoring. The latter is also used for determining the necessity of histidine, glutamine, and asparagine flips, histidine protonation states and hydroxyl orientations of water in close contact with the protein. Figure 8b shows the flipped and un-flipped state of glutamine 90 in the PDB entry 1REI [51].

#### Display of protein electron density maps

When reading a file from the PDB rather than a locally stored PDB file, the four-letter PDB identifier is stored by Molden. On clicking the “Elec. Dens. Map” button, this identifier is used to automatically retrieve the corresponding omap file from the electron density server at Uppsala University (http://eds.bmc.uu.se/eds/) [52]. After the file is read, a window will pop up, in which the user can specify the electron density contour level. For the sake of clarity, the rendered electron density volume can be clipped in three directions. Figure 8c displays, as an example, the electron density for the PDB file 2ETE [53].

#### Ambfor and Ambmd: protein geometry optimisation and protein dynamics

Ambfor was designed as an energy minimisation tool and Ambmd as a stand-alone Molecular Dynamics program. Both programs were developed to be run from within the Molden interface and their output can be visualised in real time in Molden. For small molecules the GAFF [41] force field is used and for proteins the AMBER force field [44]. Both force fields can be used together so that proteins and their ligand(s) can be optimised simultaneously. A parallelised version for both programs was developed with the help of the Message Passing Interface (MPI) library [54]. Ambfor makes use of the limited memory BFGS method [55] for optimisation of proteins. For small molecule optimisation a Powell-Beale conjugate gradient method is employed [42]. Both Ambfor and Ambmd use a damped shifted force protocol [56] that greatly reduces the number of pairwise interactions that have to be calculated. The Berendsen thermostat [57] is used to keep MD simulations at a constant temperature by scaling the velocities of the particles. By default the ff99sb extension of AMBER version 99 [58] is used. All other commonly used MD facilities such as placing the molecule in a water box, boundary conditions, temperature and run time selection, etc., have been implemented too. Energy minimisations and molecular dynamics simulations require that all molecules are chemically correct, which often requires that all hydrogens, and sometimes also some protein side chain C-, O-, and N-atoms are added.

### Ambfor validation

Since the AMBER and GAFF force field have been extensively validated previously [59], we merely need to validate the implementation of these force fields in Molden.

The Ambfor module is validated by comparing root mean square deviations between protein data bank non-hydrogen coordinates and Ambfor optimised coordinates of 26 protein ligand complexes, at different gradient tolerances (see Table 5). The force field optimisations were performed while keeping the protein rigid and the ligand fully flexible. Charges were applied to the ligand through the default charge scheme in Molden for ligands in proteins [60].

**Table 5:** Root mean square deviations between optimised coordinates and PDB coordinates for 26 protein-ligand complexes at two different gradient tolerances

PDB entry	PDB ligand code	RMSD Av. gradient < 0.5 (kcal/mol)/Angstrom	RMSD Av. gradient < 0.1 (kcal/mol)/Angstrom
2CEJ	1AH	0.040	0.155
4HYF	1AK	0.061	0.211
4NAN	2JM	0.067	0.648
4ANP	3QI	0.028	0.104
4WLB	3QQ	0.021	0.133
3QTI	3QT	0.025	0.051
5CBJ	4ZD	0.031	0.444
5CCR	4ZT	0.058	0.154
5CC3	4ZU	0.043	0.140
5CCN	4ZZ	0.073	0.163
2VIN	505	0.020	0.197
5CEO	50D	0.038	0.174
4FTR	5HK	0.029	0.223
5JJS	6L2	0.043	0.147
5JN2	6LO	0.040	0.393
5JMS	6LP	0.040	0.181
3HMM	855	0.034	0.102
2R7L	AMZ+ATP	0.162	0.953
2UZN	C96	0.198	0.386
1IEL	CAZ	0.062	0.308
1PPP	E6C	0.418	0.923
1LEV	F6P	0.049	0.440
4DFR	MTX	3.493	3.451
1OWY	PRY	0.253	0.503
5CCL	SAM	0.040	0.161
3VHU	SNL	0.078	0.160

The average RMSD at gradient tolerance 0.5 (kcal/mol)/Angstrom is 0.209 Angstrom and at gradient tolerance 0.1 (kcal/mol)/Angstrom is 0.420 Angstrom. The one exceptionally bad case (MTX in 4DFR) is explained by the fact that MTX sticks out of the DFR pocket and makes symmetry contacts in the crystal. Molden cannot yet automatically include such symmetry contacts. When the symmetry related DFR copy is added manually, the two RMSD values become 0.118 and 0.264, respectively.

#### Addition of hydrogens to ligands

‘wget’ can be used to retrieve information about missing hydrogens of ligands in a PDB entry by downloading a version of the ligand with hydrogens added from the EBI’s PDBECHEM database [19] (ftp://ftp.ebi.ac.uk/pub/databases/msd/pdbechem/files/cml).

#### Fixing incomplete residues

Crystallography does not always reveal the position of all atoms in a protein. Especially the, often mobile, extremities of Glu, Gln, Arg, and Lys, occasionally are missing in PDB file. In case of missing atoms it is possible to either automatically complete the residue, or to use the Z-matrix editor to do this manually. Many protons are not mobile with respect to the heavy atom they are bound to (e.g. the protons on a phenyl ring). These so-called riding protons are placed using a dictionary of proton positions. The positions of other protons can be determined using Ambfor.

#### Protein-specific visualisation facilities

**Fig. 9:**
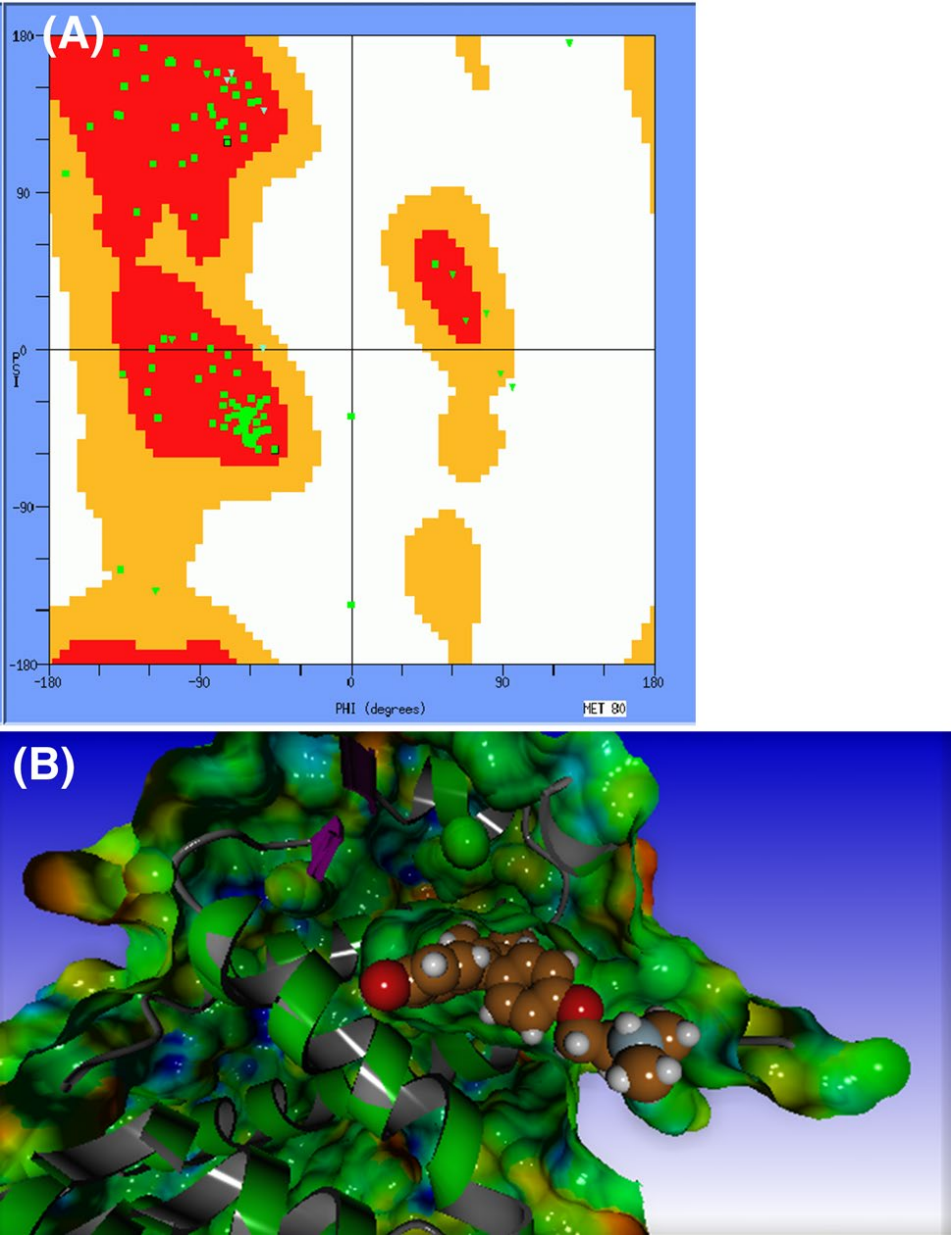
**a** Ramachandran plot with favoured (*red*) and allowed (*orange*) regions of backbone torsion angles. **b** Clipped solvent accessible surface of PDB entry 3ERT [64] (with the electrostatic potential mapped onto it) exposing the OHT ligand

Molden has a large series of protein visualisation facilities available. It can, for example produce Ramachandran plots [61] (see Fig. 9a). Backbone secondary structure elements such as alpha helix and β-sheets tend to be contained in preferred conformational regions of the plot according to Lovell et al. [62]. Residues that fall outside these regions can be flagged as outliers. By clicking a green dot in the plot, the corresponding amino acid is displayed in solid sphere representation in the main window.

Solvent accessible surface areas can be produced using the external program Surf [63] that is distributed together with Molden. Optionally, the electrostatic potential calculated from point charges associated with the AMBER force field can be mapped onto this surface. Surfaces can be clipped to reveal the interaction of a ligand with the protein surface, which is especially useful when the ligand is deeply buried within the protein. Figure 9b shows the clipped solvent accessible surface of the PDB entry 3ERT [64], with the electrostatic potential mapped onto it, exposing the OHT ligand.

## Conclusions

Fifteen years after the release of Molden 1.0 the program is still being used by thousands of researchers around the world. In these 15 years the original set of options has been maintained and extended. The scope of Molden has also been broadened by adding many drug-design related facilities. We have described a series of improved or novel facilities. The list of novel facilities, though, is much longer than we have space for in this article. Examples are: protein structure alignment and superposition, creation of movies, support for Mopac .aux, and VASP POSCAR files, interactive generation of crystals from single molecules, reading .sdf files, making snapshots and movies, interfaces to the universal file converter openbabel [65] and the pharmacophore search machine pharmer [66]. We hope that Molden 2.0 will contribute as much to the world of small molecule science as did Molden 1.0. The release of Molden 3.0 is planned less than 15 years from now. Molden 3.0 will broaden its scope further by expanding in the direction of drug design, incorporating functionalities such as conformational analysis and docking of small molecules. Interfacing Molden with more key softwares in the QM–MM field has started. The Ambfor crystal optimiser module will be improved by use of Ewald summation of long range interactions.
